# Malaria Cytoskeletal Proteins Require Alveolin–Alveolin Interactions for Differential Localization: Recruitment and Organization of Alveolin Proteins

**DOI:** 10.1155/cmi/4530231

**Published:** 2025-07-03

**Authors:** Ana Karla Cepeda Diaz, Peter S. Back, Sreelakshmi K. Sreenivasamurthy, Jeffrey D. Dvorin

**Affiliations:** 1 Division of Infectious Diseases, Boston Children’s Hospital, Boston, Massachusetts, USA; 2 Biological and Biomedical Sciences, Harvard Medical School, Boston, Massachusetts, USA; 3 Department of Pediatrics, Harvard Medical School, Boston, Massachusetts, USA

## Abstract

The alveolins are a family of intermediate filament-like proteins that form cytoskeletal structures in both free-living and parasitic members of the alveolate kingdom. Despite their important functions, the alveolins’ biochemical properties and organizing principles are still poorly understood. Here, we characterize four alveolins of *Plasmodium falciparum*, the deadliest malaria parasite, to understand how alveolin domains mediate protein–protein interactions and highly specific recruitment to substructures of the cytoskeleton. Unexpectedly, we uncover variable dependence on alveolin domains for each substructure rather than an overarching mechanism. While *Pf*IMC1e requires 1f to be sequentially recruited to the basal complex, *Pf*IMC1c and *Pf*IMC1g do not require interactions with each other to localize properly to the inner membrane complex. Moreover, alveolin domains are not interchangeable—they contain unique signatures for specialized localization. Finally, we identify a region outside the alveolin domain of *Pf*IMC1e that is important for basal complex recruitment. These results provide direct evidence that alveolin domains mediate both alveolin–alveolin interactions and compartment-specific localization.

## 1. Introduction

*Plasmodium* parasites, the causative agents of malaria, have specialized cytoskeletal structures that are essential to support the parasite’s complex lifecycle. One of these structures is the inner membrane complex (IMC), which serves a cytoskeleton-like function in conjunction with the mesh-work of proteins that lies attached to its cytoplasmic face, the subpellicular network (SPN) [1]. These highly divergent structures are a distinguishing feature of alveolate cytoskeletal systems and absent from metazoans, including mammals [2–4]. The SPN is a series of filaments that are composed of a family of proteins called alveolins [1, 5, 6]. Studies in both *Plasmodium* and its relative *Toxoplasma* have demonstrated that alveolin knockouts lead to defects in cell shape, motility, and infectivity in addition to compromised tensile strength [3, 7–11]. Thus, the SPN filaments are thought to provide mechanical stability, impacting many of the parasite’s critical processes including motility, invasion, and cell division.

All alveolins contain at least one valine- and proline-rich repeat domains (called the alveolin or IMCp domain) with a consensus heptad repeat sequence of EKIVEVP [12]. The regions outside this domain vary greatly in size and do not have conserved features other than predicted posttranslational modification sites [12, 13]. There are 13 alveolins (IMC1a–m) currently annotated in the *Plasmodium* genome. Despite their “IMC” nomenclature, *Plasmodium* alveolins have distinct spatial organizational patterns, localizing to either the IMC or the basal complex (BC) [8, 10, 11, 14–17]. The BC is a multiprotein structure that is believed to guide the formation of parasite membranes during cell division, another key component of the cytoskeleton, and likely mediates abscission during cytokinesis [18]. It remains unclear how alveolins achieve these specific spatial patterns or whether alveolins in different compartments hold different biological functions.

The alveolin domain has also been shown to be necessary and largely sufficient for trafficking, often hypothesized to mediate protein–protein interactions for filament formation [19]. Deleting the alveolin domain of PbIMC1h caused loss of protein staining at the IMC in the rodent malaria parasite *Plasmodium berghei*, and similar observations have been made in the free-living ciliate *Tetrahymena* [20, 21]. In the parasitic relative *Toxoplasma*, the alveolin domains of TgIMC3, 6, and 8 on their own showed compartment-specific localization [19, 22]. To achieve wild-type localization, however, TgIMC8 required N- and/or C-terminal sequences, implying important functions also lie outside the alveolin domain [19]. Adding to the complexity, many nonalveolin binding partners have been proposed to participate in the recruitment and stabilization of the alveolin network at the IMC. The most notable examples are the GAPMs in *Plasmodium* and ILP1 in *Toxoplasma*, neither of which contains alveolin domains but was demonstrated to bind alveolin proteins [7, 23, 24].

Overall, much remains unknown about the mechanisms behind alveolin function, filament formation, and recruitment to specific cell compartments. Studies thus far have suggested a great diversity in individual alveolin function, localization, and timing of expression during the parasite lifecycle. This diversity indicates that new alveolins in new parasites and lifecycle stages must be individually interrogated. It also motivates us to dissect the primary sequence of these proteins in more detail to identify the molecular signatures of these phenotypes. In this study, we examine the role of the alveolin domain in facilitating interactions between alveolin proteins. We also identify a region outside the alveolin domain, the noncanonical repeat (NCR) domain, that is required for localization. Of the 13 annotated alveolins in the genome, *Plasmodium falciparum* only expresses four during the asexual blood stage, two that localize to the IMC, and two that localize to the BC. We take advantage of this minimal number to assess the contribution of the alveolin domain to filament formation and compartment-specific recruitment.

## 2. Results and Discussion

**Figure 1:**
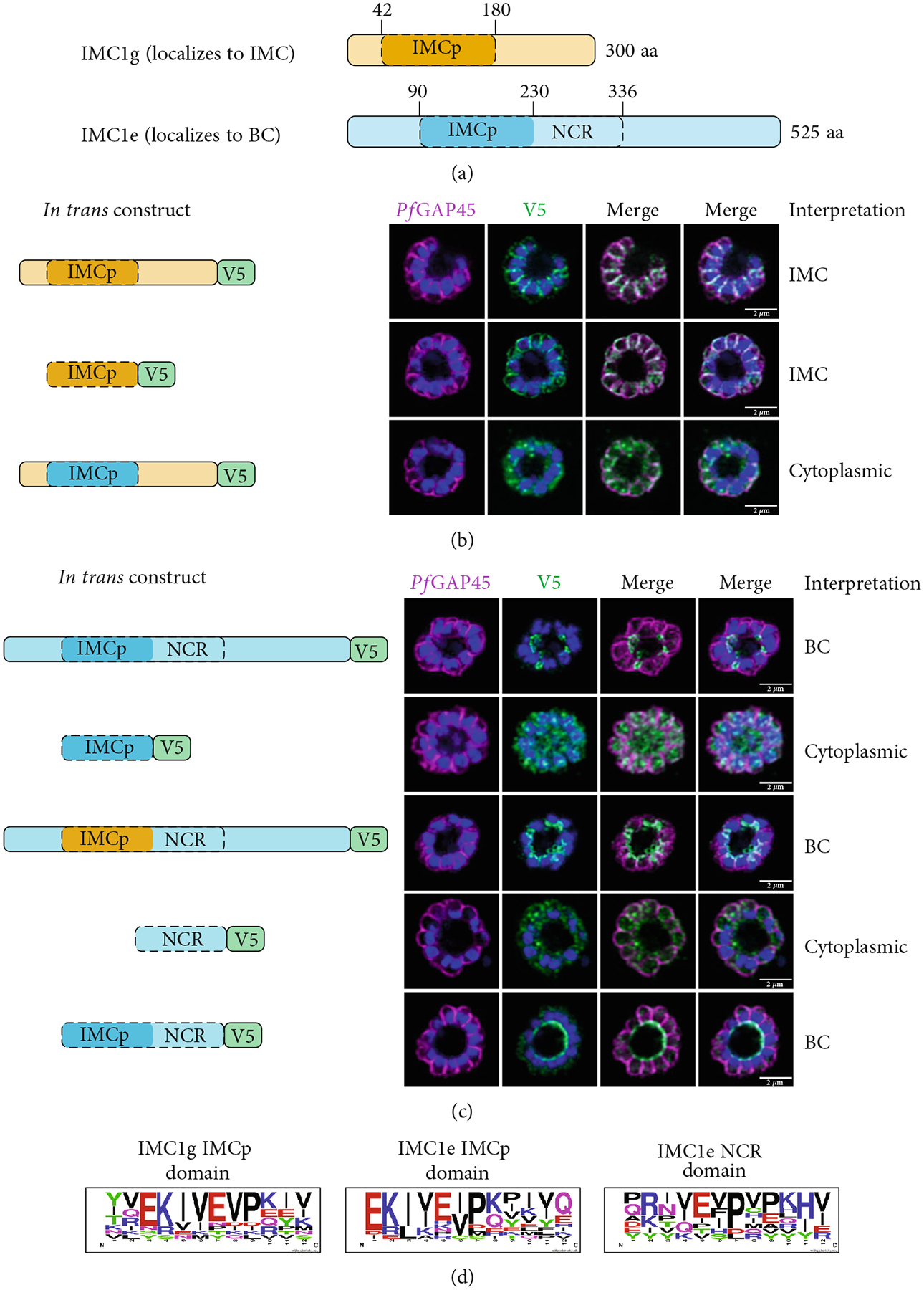
The alveolin domain is not always sufficient for recruitment to the IMC. (a) Diagram of *Pf*IMC1g (orange) and 1e (blue). Predicted alveolin domain (IMCp) shaded in dark hue. Repeat region outlined with dashed line. (b, c) Airyscan superresolution images of IFA showing localizations of V5-tagged *in trans* constructs. A diagram of each construct is shown on the left, and our interpretation of localization is noted on the right. *Pf*GAP45 (shown in magenta) is used as an inner membrane complex marker. 4′,6-Diamidino-2-phenylindole (DAPI) stains parasite DNA (shown in blue). (b) *Pf*IMC1g constructs including full-length (top), alveolin domain only (middle), and hybrid constructs (bottom). (c) *Pf*IMC1e constructs including (from top to bottom) full-length, alveolin domain only, hybrid, NCR region only, and alveolin domain with NCR region constructs. (d) Consensus sequence of repeats in the *Pf*IMC1g alveolin domain (left), *Pf*IMC1e alveolin domain (middle), and NCR region (right). Scale bars = 2 *μ*m.

In order to test the determinants of localization for *Plasmodium* proteins, we generated *in trans* expression constructs containing either selected domains of interest or full-length sequence of *Pf*IMC1g, an IMC alveolin, and *Pf*IMC1e, a BC alveolin (Figure 1; Supporting Information 2: Table S1) [8]. These constructs were all tagged with either single V5 or smV5 tags [25] and expressed from the *Pfbleb* locus, a schizont-stage BC protein that is dispensable for asexual development [26]. The *in trans* constructs replace the coding region of *Pf*BLEB and are expressed from its endogenous promoter, largely matching the level and timing of expression for the alveolins. To mitigate potential toxicity from protein overexpression, we inserted these constructs in an inversion-based Cre-inducible system. Briefly, the ORF of our *in trans* constructs was inverted relative to the *Pfbleb* promoter and flanked by two pairs of heterospecific *lox* sites (*loxP511* and *loxN*) oriented towards each other (see Supporting Information 1: Figure S1A for diagram). These parasite lines also contained a dimerizable Cre (DiCre) recombinase which becomes active upon the addition of rapamycin (RAPA). In theory, this would enable inducible expression of our constructs by irreversibly flipping the ORF in frame of the *Pfbleb* promoter. However, we found that leakiness in the system led to flipping even without RAPA, resulting in comparable expression levels between uninduced and induced conditions (Supporting Information 1: Figure S1B). Fortunately, these constructs showed no toxicity despite the *trans*-expression, allowing us to evaluate our constructs *in trans*. We harvested late (46–48 h postinfection, hpi) schizonts expressing these constructs and used immunofluorescence assays (IFAs) against V5 to visualize the localization of our tagged constructs and against *Pf*GAP45 to visualize the IMC of parasites [27].

### 2.1. The Alveolin Domain Is Not Always Necessary and Sufficient for Recruitment to the IMC.

We first tested whether the alveolin domain of *Pf*IMC1g was sufficient for its recruitment to the IMC. The alveolin domain was identified using InterPro and consisted of amino acids 42–180 of the protein (Figure 1a) [28]. Constructs expressing the alveolin domain of *Pf*IMC1g on its own were properly localized, akin to the full-length protein (Figure 1b). We conducted a similar analysis with *Pf*IMC1e. The *Pf*IMC1e alveolin domain, again identified using InterPro, consisted of amino acids 90–230 (Figure 1a). While full-length *Pf*IMC1e localized to the BC, the alveolin domain on its own showed cytoplasmic staining (Figure 1c). To ensure that the mislocalized constructs are stably expressed, we performed western blots (Supporting Information 1: Figure S1A–D). Untagged 3D7-DiCre parasites showed no staining when probed with anti-V5 antibody, as expected (Supporting Information 1: Figure S1C). The IMC1g alveolin domain (Supporting Information 1: Figure S1D) and the IMC1e alveolin domain (Supporting Information 1: Figure S1E) both exhibited robust expression at the expected molecular weight despite only the former localizing properly by IFA. Thus, unlike the alveolin domain of *Pf*IMC1g, the alveolin domain of *Pf*IMC1e was not sufficient for recruitment to the BC (or the IMC).

Next, we tested the necessity of these alveolin domains for recruitment to the IMC and BC. Given the size of the alveolin domain, simple deletions may cause protein misfolding or instability, so we instead created hybrid constructs that replaced the alveolin domain with a different sequence of similar length and amino acid composition.

For *Pf*IMC1g, we replaced amino acids 42–180 with the *Pf*IMC1e alveolin domain, which is similar in length and amino acid composition to the *Pf*IMC1g domain but does not have IMC-targeting properties. This construct was unable to localize to the IMC despite preserving the *Pf*IMC1g N- and C-termini (Figure 1b). Western blots revealed that this hybrid protein is expressed at the expected size, though the intensity is weak, suggesting low abundance (Supporting Information 1: Figure S1F). While we cannot formally exclude the possibility that this construct is unstable, the alveolin domain of *Pf*IMC1g appears to be both necessary and sufficient for IMC recruitment.

For *Pf*IMC1e, we replaced amino acids 90–230 with the *Pf*IMC1g alveolin domain, which does have IMC-targeting properties. Surprisingly, this construct primarily localized to the BC with some minor staining at the IMC (Figure 1c). Thus, the primary targeting sequence that recruits *Pf*IMC1e to the BC must lie outside of its alveolin domain.

It is not obvious what marks the difference between the alveolin domains of *Pf*IMC1g and *Pf*IMC1e. Both domains are the same length (~140 aa) and consist of 13 valine- and proline-rich repeats with the characteristic consensus heptad of the alveolins (EKIVEVP) (Figure 1d). However, the position of this heptad within the 12 amino acid repeats is not consistent between *Pf*IMC1g and *Pf*IMC1e. The heptad begins at Position 3 in *Pf*IMC1g and Position 1 in *Pf*IMC1e (Supporting Information 1: Figure S2). It is unclear to what extent this positioning has any biological relevance.

### 2.2. PfIMC1e Contains an Additional Repeat Domain With BC-Targeting Properties.

We further analyzed the *Pf*IMC1e sequence, which revealed an additional stretch of valine- and proline-rich repeats not identified as an alveolin domain by InterPro [29, 30]. This region spans amino acids 230–336 and contains an additional nine repeats but does not maintain the canonical alveolin consensus sequence (Figure 1d; Supporting Information 1: Figure S2). We have termed this region the non-canonical repeat (NCR) region (Figure 1a).

We tested whether the *Pf*IMC1e NCR region could localize to the BC on its own or when coupled with the canonical alveolin domain identified by InterPro. On its own, the NCR region was not able to localize to the BC, despite robust expression by western blot (Figure 1c; Supporting Information 1: Figure S1G). However, when the NCR was coupled with the alveolin domain, the resulting construct was localized to the BC (Figure 1c). So, while neither the alveolin domain nor the NCR region of *Pf*IMC1e is, on their own, sufficient for BC localization, together they form a minimal domain for localization.

### 2.3. PfIMC1e and PfIMC1f Are Dispensable and Sequentially Recruited to the BC.

We previously showed that *Pf*IMC1f was dispensable in the asexual blood stages by direct knockout [8]. Since we failed to directly knock out *Pf*IMC1e, here, we employed an inducible knockout approach to assess its essentiality. This line used *loxP* sites and DiCre to excise the *Pf*IMC1e coding sequence upon RAPA addition [8, 31], generating the *Pf*IMC1e^iKO^ line.

**Figure 2:**
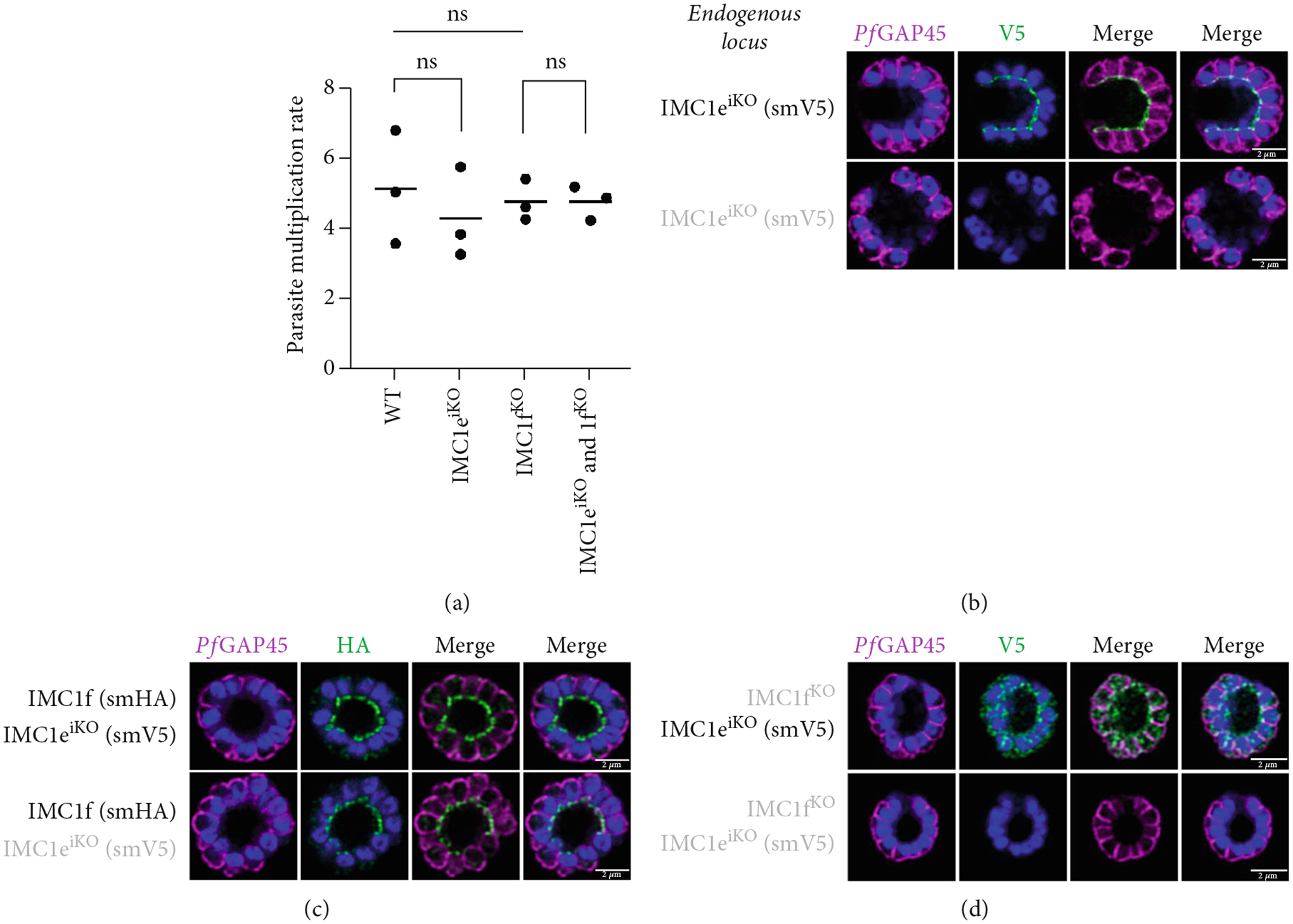
*Pf*IMC1e and 1f are dispensable and recruited to the basal complex sequentially. (a) From left to right, the replication of WT, *Pf*IMC1e^iKO^ (+RAPA), *Pf*IMC1f^KO^, and *Pf*IMC1f^KO^ + *Pf*IMC1e^iKO^ (+RAPA) parasites as measured by flow cytometry. The parasite multiplication rate was calculated as follows: Day 4 parasitemia (% infected RBCs)/Day 2 parasitemia. Three biological replicates and mean were plotted. (b–d) All IFAs show the endogenous loci of parasite strain on the left where black text indicates the presence and gray text indicates the absence of the protein. IFAs are costained with *Pf*GAP45 (magenta) as an IMC marker and DAPI as DNA stain (blue). Scale bars = 2 *μ*m. (b) IFA showing localization of endogenous smV5-tagged *Pf*IMC1e in DMSO (black text, top panel) and in RAPA (gray text, bottom panel) conditions. (c) IFA showing localization of endogenous smHA-tagged *Pf*IMC1f in the presence and absence of its partner *Pf*IMC1e^iKO^. DMSO condition is shown in black text, top panel; RAPA condition is shown in gray text, bottom panel. (d) IFA showing localization of endogenous smV5-tagged *Pf*IMC1e when its partner *Pf*IMC1f is knocked out. Top panel: DMSO condition where *Pf*IMC1e-smV5 is present. Bottom panel: RAPA condition where *Pf*IMC1e-smV5 is absent, effectively a double knockout.

Parasite replication, as measured by flow cytometry, was unaffected in *Pf*IMC1e-deficient (+RAPA) conditions when compared to its wild-type (+DMSO) control (Figure 2a). As expected, wild-type (+DMSO) parasites showed *Pf*IMC1e staining at the BC, while *Pf*IMC1e-deficient (+RAPA) parasites showed no staining of *Pf*IMC1e at the BC (Figure 2b). These parasites also lacked any obvious morphological abnormalities.

Since *Pf*IMC1e and *Pf*IMC1f share a BC localization and are individually dispensable, we assessed whether there was redundancy between the two proteins. We generated a line where *Pf*IMC1f is directly knocked out and where *Pf*IMC1e can be conditionally knocked out. Once more, we saw no difference in parasite replication when both proteins were absent (Figure 2a).

To assess whether *Pf*IMC1e and *Pf*IMC1f showed dependence on each other for recruitment, we performed IFAs on our knockout parasites to visualize the localization of one protein in the absence of the other. When *Pf*IMC1e^iKO^ is knocked out, the localization of endogenous *Pf*IMC1f, visualized via an epitope tag on the endogenous protein, remains at the BC (Figure 2c). Moreover, western blots show that *Pf*IMC1f levels remain comparable when *Pf*IMC1e^iKO^ is knocked out as well (Supporting Information 1: Figure S1H). However, when *Pf*IMC1f is knocked out, *Pf*IMC1e is absent from the BC and instead shows cytoplasmic staining (Figure 2d). Western blots show that mislocalized *Pf*IMC1e remains stably expressed (Supporting Information 1: Figure S1I). This suggests that these alveolins are recruited to the BC sequentially, with *Pf*IMC1f recruiting *Pf*IMC1e. Furthermore, it means that the *Pf*IMC1f knockout, on its own, produces a double KO-like effect by preventing the recruitment of *Pf*IMC1e, reinforcing our conclusion that these two proteins do not show functional redundancy.

### 2.4. The Alveolin Domain Recruits PfIMC1g to the IMC by Facilitating Interactions With PfIMC1c.

We then tested whether alveolin–alveolin interactions are also important for recruitment to the IMC. We expressed our *Pf*IMC1g alveolin domain construct in lines where the endogenous *Pf*IMC1g or its putative partner *Pf*IMC1c could be depleted using the TetR-DOZI inducible knockdown (iKD) system [32, 33].

**Figure 3:**
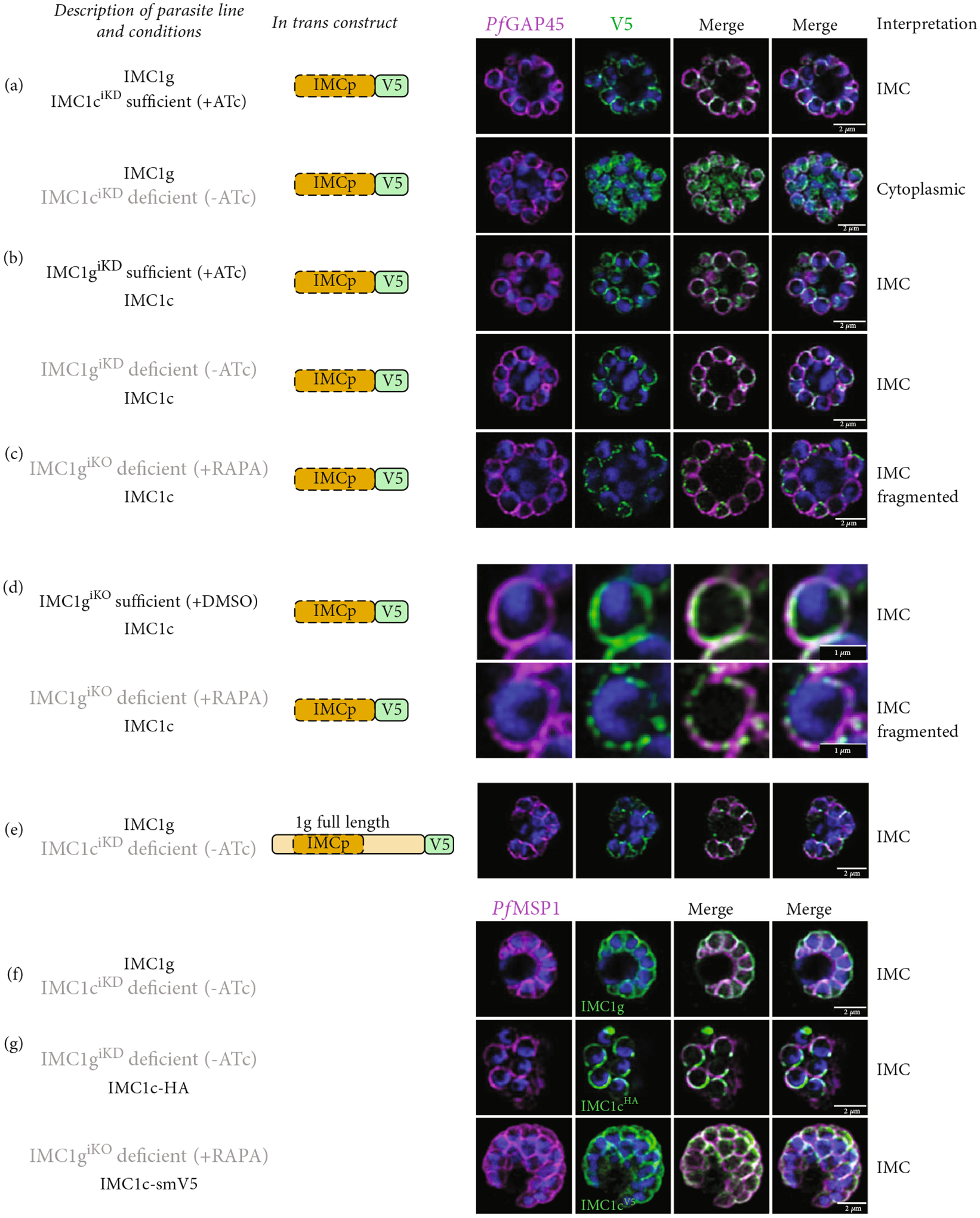
The *Pf*IMC1g alveolin domain requires homo- and heterointeractions for recruitment. All IFAs include a description of the endogenous locus, the *in trans* construct, and interpretation of the localization. In the description, just the protein name indicates an unmodified locus, black text indicates the presence of the protein, and gray text indicates the absence of the protein. IFAs are costained with *Pf*GAP45 as an IMC marker or *Pf*MSP1 as a plasma membrane marker and DAPI for parasite DNA. (a) IFA of the V5-tagged *Pf*IMC1g alveolin domain in the presence and absence of its partner *Pf*IMC1c^iKD^. (b) IFA of the V5-tagged *Pf*IMC1g alveolin domain in the presence and absence of endogenous *Pf*IMC1g^iKD^, by conditional knockdown. (c) IFA of V5-tagged *Pf*IMC1g alveolin domain in the absence of endogenous *Pf*IMC1g^iKO^, by conditional knockout. (d) Zoomed-in view of one merozoite where the *Pf*IMC1g alveolin domain construct appears to localize in a fragmented pattern upon conditional knockout of endogenous *Pf*IMC1g^iKO^. (e) IFA of the full-length *Pf*IMC1g construct in the absence of its partner *Pf*IMC1c. (f) IFA of endogenous *Pf*IMC1g (primary antibody) when its partner *Pf*IMC1c^iKD^ is knocked down. (g) IFA of endogenously tagged *Pf*IMC1c in the absence of its partner *Pf*IMC1g, either by conditional knockdown (iKD, top panel) or by conditional knockout (iKO, bottom panel). Scale bars = 2 *μ*m.

With endogenous *Pf*IMC1c^iKD^ present (+anhydrotetracycline (ATc)), the *Pf*IMC1g alveolin domain was localized properly, as expected. However, when endogenous *Pf*IMC1c^iKD^ is knocked down (−ATc), the *Pf*IMC1g alveolin domain loses its IMC recruitment and instead becomes cytoplasmic (Figure 3a). Western blots indicate that the construct remains stably expressed despite its mislocalization (Supporting Information 1: Figure S1D). This is a confirmation that *Pf*IMC1g and 1c are indeed interacting partners and that their interaction requires the alveolin domain. On the other hand, when endogenous *Pf*IMC1g is either present (+ATc) or knocked down (−ATc), the *Pf*IMC1g alveolin domain remains at the IMC (Figure 3b). To account for the possibility that residual amounts of *Pf*IMC1g was responsible for IMC recruitment, we repeated this experiment in an *Pf*IMC1g inducible knockout line (IMC1g^iKO^), utilizing the insertion of *loxP* sites flanking the endogenous *Pfimc1g* locus [8, 31]. Upon the knockout of endogenous *Pf*IMC1g^iKO^, the *Pf*IMC1g alveolin domain was again localized to the IMC (Figure 3c). This suggests that the *Pf*IMC1g alveolin domain is recruited to the IMC through a pathway that does not require self-interactions with full-length *Pf*IMC1g but does require interactions with *Pf*IMC1c. Interestingly, when *Pf*IMC1g was knocked down or knocked out, staining of the *Pf*IMC1g alveolin domain at the IMC became fragmented (Figure 3d). Thus, while the alveolin domain of *Pf*IMC1g absolutely requires *Pf*IMC1c for localization, additional interactions with full-length *Pf*IMC1g may stabilize the localization.

### 2.5. PfIMC1c Depletion Does Not Affect Recruitment of Full-Length PfIMC1g.

A sequential recruitment pathway in which *Pf*IMC1c is recruited to the IMC first and then recruits *Pf*IMC1g would explain the dependence of *Pf*IMC1g’s alveolin domain on *Pf*IMC1c for recruitment. To test this possibility, we looked at whether *Pf*IMC1c knockdown had any effect on the localization of full-length *Pf*IMC1g.

To our surprise, depleting *Pf*IMC1c^iKD^ did not have any effect on the recruitment of *in trans* (Figure 3e) or endogenous full-length *Pf*IMC1g (Figure 3f). This suggests that the N- and C- termini of *Pf*IMC1g contain domains that, when combined with its alveolin domain, are sufficient for IMC localization. Since these experiments were conducted in a knockdown background, we cannot exclude the possibility that residual *Pf*IMC1c is sufficient to recruit full-length *Pf*IMC1g. However, neither depletion of *Pf*IMC1g^iKD^ nor the knockout of *Pf*IMC1g^iKO^ disrupts the recruitment of endogenous *Pf*IMC1c (Figure 3g). So, an alveolin-independent recruitment pathway must exist, at least to recruit *Pf*IMC1c, if not to recruit both alveolins.

### 2.6. Predicted Structure of Alveolins Provides Insights Into Multimerization and Role of the Alveolin Domain.

To further explore the hypothesis that *Pf*IMC1c and 1g are interacting partners, we compared their predicted structures as monomers with their predicted structures as multimers using AlphaFold and AlphaFold Multimer [34, 35]. On their own, *Pf*IMC1g and 1c do not display much ordered secondary structure (Supporting Information 1: Figure S3A and B). However, when they are modeled as multimers with each other, they form a twisted *β*-ribbon structure (Supporting Information 1: Figure S3C). We also tested our theory that *Pf*IMC1e needs both its alveolin domain (or an equivalent domain) and its NCR region to adopt a conformation that is stable and can interact with its BC partners. When modeled individually, the NCR region and alveolin domain of *Pf*IMC1e are disordered (Supporting Information 1: Figure S3D and E)—much like *Pf*IMC1g and 1c. However, when modeled together, they form a *β*-ribbon like the one formed by *Pf*IMC1g and 1c (Supporting Information 1: Figure S3F). So, alveolin domains are likely disordered or misfolded as monomers and require interactions with additional monomers or domains to be able to adopt a stable conformation that allows them to bind their partner proteins.

**Figure 4:**
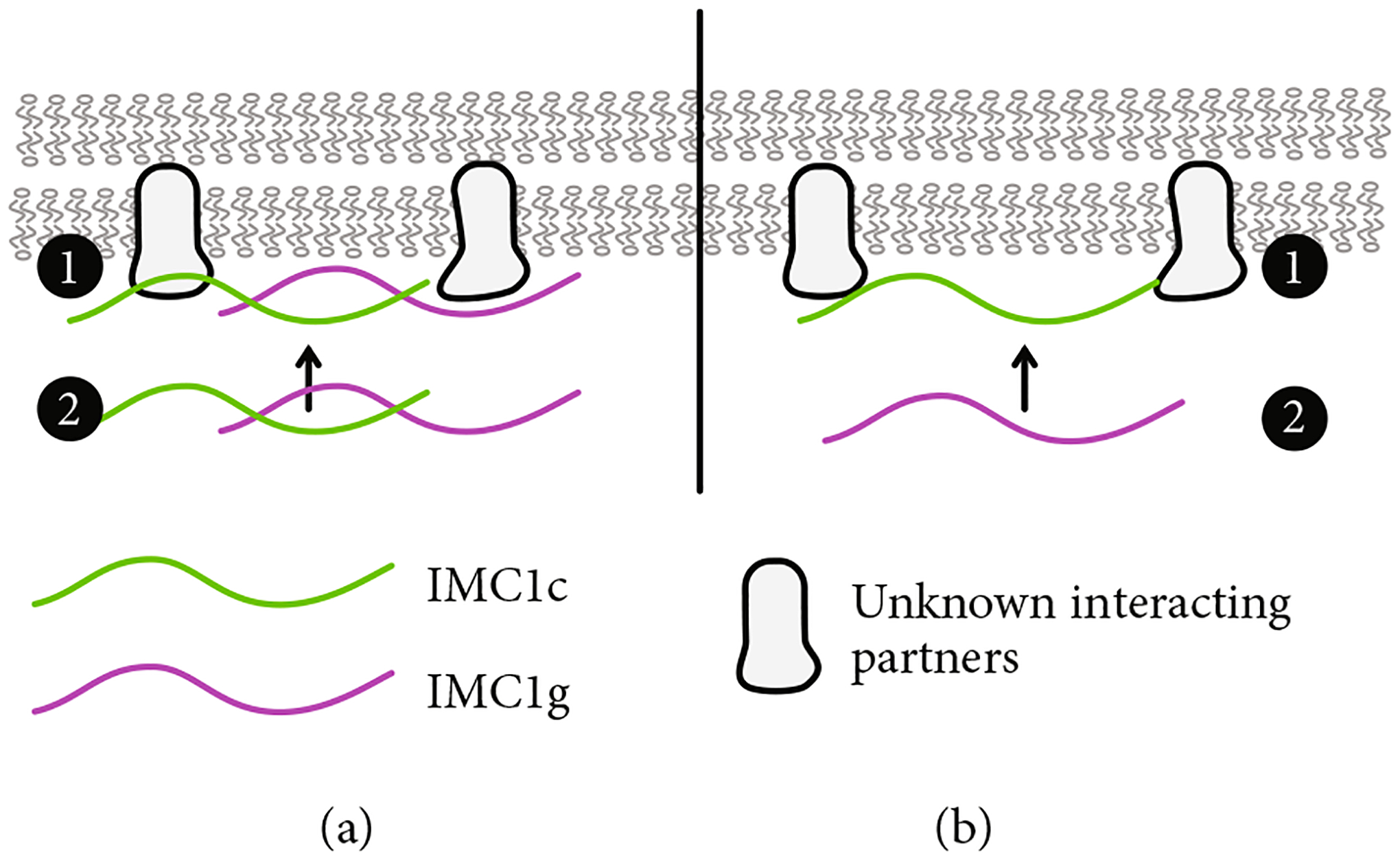
Model of potential alveolin recruitment pathways for *Pf*IMC1g and 1c. (a) *Pf*IMC1g and 1c are recruited through both alveolin-dependent and alveolin-independent pathways. The alveolin-independent pathway could be facilitated by palmitoylation or a yet-to-be-identified interacting partner. Alveolin–alveolin interactions stabilize monomers into composite filaments at the IMC (1). *Pf*IMC1g and 1c at the IMC recruit other *Pf*IMC1g and 1c monomers to form filaments (2). (b) Sequential recruitment pathway in which *Pf*IMC1c is recruited directly to the IMC by palmitoylation or partner proteins (1) and *Pf*IMC1g is recruited by forming filaments with *Pf*IMC1c.

If *Pf*IMC1g and 1c are indeed unstructured as monomers, then a sequential recruitment model seems less likely. *Pf*IMC1e, on the other hand, achieves the *β*-ribbon conformation using intramolecular interactions and can also form a *β*-ribbon when modeled with the *Pf*IMC1f alveolin domain, which is in line with a model of sequential recruitment (Figure 4 and Supporting Information 1: Figure S3G). Our AlphaFold models suggest that the *β*-ribbon requires a specific partner and cannot form with just any other alveolin domain. While models of *Pf*IMC1g dimerizing with 1c have high per-residue model confidence scores (pLDDT), our modeling does not support the ability of *Pf*IMC1g to form dimers with itself or *Pf*IMC1e. All such multimer predictions are largely unstructured and have low (pLDDT < 50) confidence (Supporting Information 1: Figure S3H and I). Models of the NCR region, on the other hand, suggest that it can attain an intramolecular *β*-ribbon conformation with both the *Pf*IMC1e (70 < pLDDT < 95) and 1g (50 < pLDDT < 80) alveolin domains, though interactions with the *Pf*IMC1g domain, as evaluated by model confidence, are less favorable (Supporting Information 1: Figure S3J). This is consistent with our IFA data which shows that the *Pf*IMC1g alveolin domain can replace that of *Pf*IMC1e without causing a loss in BC localization.

### 2.7. Alveolin–Alveolin Interactions Are Necessary for Filament Formation and Compartment Specificity.

In this work, we assessed the role of the alveolin domain for the function and localization of asexual blood-stage alveolins. This is the first investigation to determine how alveolins localize to specific subcompartments in *Plasmodium*, yielding multiple unexpected results including direct evidence of alveolin–alveolin dependence.

We show that the alveolin domain of *Pf*IMC1g is sufficient for recruitment to the IMC in a WT background. However, when *Pf*IMC1c is depleted, the alveolin domain of *Pf*IMC1g is no longer sufficient for recruitment. This confirms that *Pf*IMC1g and *Pf*IMC1c are interacting partners and further maps that interaction to the alveolin domain. Surprisingly, the recruitment of the full-length *Pf*IMC1g was not affected by the depletion of *Pf*IMC1c. *Pf*IMC1c recruitment was also unaffected by the absence of *Pf*IMC1g.

Based on this data, we propose two possible models for *Pf*IMC1g recruitment to the IMC (Figure 4). In the first model, *Pf*IMC1g and 1c are recruited through both alveolin-dependent and alveolin-independent pathways (Figure 4a). The alveolin-independent pathway would likely require the N- and C-termini of *Pf*IMC1g and could be facilitated by palmitoylation or a yet to be identified interacting partner. Then, the alveolin–alveolin interactions we identified likely stabilize monomers of *Pf*IMC1g and 1c into filaments at the IMC, meaning that if one is already present, the other can continue to localize to the IMC. The role of palmitoylation is likely since both *Pf*IMC1g and *Pf*IMC1c were palmitoylated in a genome-wide “palmitome” experiment [36]. In addition, *Pb*IMC1g in the rodent malaria was shown to require palmitoylation for its IMC localization during the asexual blood stages [37]. The role of nonalveolin proteins is also likely. From previous work in *Toxoplasma* [22, 24] and coimmunoprecipitation (co-IP) experiments from our lab [8], we know that alveolin proteins interact with many nonalveolin proteins. To support this idea, we colocalized IMC1g with one of these nonalveolin IMC protein called PIC5, which was the top candidate in the IMC1g co-IP dataset, previously shown to localize to the IMC, and essential for proper cell division (Supporting Information 1: Figure S4) [38, 39]. IMC1g and PIC5 appear to colocalize properly, and, while these data increase our confidence in the IMC1g-PIC5 interaction, we still do not know if PIC5 is the interactor that initiates IMC1g recruitment. Thus, both palmitoylation- and nonalveolin-interacting partners likely contribute to the alveolin-independent recruitment pathway.

An alternative sequential recruitment model where *Pf*IMC1c is recruited first and then recruits *Pf*IMC1g would also fit our observations (Figure 4b). While *Pf*IMC1c depletion did not affect *Pf*IMC1g recruitment to the IMC, it is possible that the knockdown we employed for this depletion, despite being lethal, left enough residual protein to allow for recruitment of full-length *Pf*IMC1g. Thus, this sequential recruitment cannot be ruled out without a *Pf*IMC1c knockout. Furthermore, experiments exploring the dependence of the alveolin domain of *Pf*IMC1c on *Pf*IMC1g could clarify the relationship between these two proteins and how they are recruited to the IMC.

Interestingly, when *Pf*IMC1g was depleted, staining of the *Pf*IMC1g alveolin domain at the IMC became fragmented, but there was no change in *Pf*IMC1c staining. This could indicate that, while not required for recruitment, full-length *Pf*IMC1g is needed for proper architecture of the IMC, which has a downstream effect for the binding sites of the alveolin domain. It is also possible that without full-length *Pf*IMC1g, the alveolin domain is recruited less efficiently. One might imagine that fibrils formed by the alveolin domain may have a suboptimal conformation or fewer binding sites when compared to hybrid fibrils that also contain the full-length protein. A change in the architecture or the distribution of unknown binding partners besides *Pf*IMC1c could lead to a similar effect, affecting recruitment to the IMC or decreasing binding sites. So far, none of our data or modeling has provided evidence that *Pf*IMC1g is forming homodimers. However, further experiments and modeling are necessary to rule out other types of interactions including higher order multimers and fibrils containing partner proteins as well as multiple copies of *Pf*IMC1g.

The alveolin domain of *Pf*IMC1g is both necessary and sufficient for recruitment to the IMC. For *Pf*IMC1e, we identify an additional region of repeats, termed NCR region, that lies outside the alveolin domain that is important for BC recruitment. Indeed, the alveolin domain of *Pf*IMC1e is not necessary nor sufficient for its localization. However, the combination of the alveolin and NCR domain is sufficient. We hypothesize that the alveolin domain of *Pf*IMC1e acts as a stabilizing domain that allows the NCR to adopt the right conformation for BC binding. *Pf*IMC1e and 1f show a very clear sequential recruitment pattern. So it is likely that the NCR region is facilitating interactions between the two proteins. However, additional experiments are needed to determine more robustly how and under what conditions NCRs are required for recruitment.

It also remains unclear from our data what defines, functionally, the alveolin domain of a protein. The NCR region, for example, could very well be considered a part of the alveolin domain of *Pf*IMC1e if, instead of focusing on specific amino acid identities, we focused more on amino acid properties. We note that the alveolin domain of *Pf*IMC1c contains enough repeats which deviate from the canonical consensus heptad of EKIVEVP in both amino acid identity and properties that it could also be considered noncanonical. Defining the characteristics of these proteins is key to our understanding of how they function, specialize, and evolve.

The hypothesis we put forward regarding the stabilizing role of the alveolin domain of *Pf*IMC1e is consistent with our modeling of protein secondary structure using AlphaFold and AlphaFold Multimer. On their own, the alveolin domains of *Pf*IMC1e, 1g, and 1c do not display any ordered secondary structure. However, when the alveolin domain of *Pf*IMC1e is modeled alongside the NCR region, it attains a *β*-ribbon structure. The same is true when *Pf*IMC1g and 1c are modeled as a heterodimer. This supports our hypothesis that alveolin domains are likely disordered or misfolded on their own and require interactions with additional monomers or domains to be able to bind their partner proteins and localize properly. While our modeling data suggests that these interactions must occur with specific partner proteins, we have not modeled multimers beyond dimers, and we have only tested a few putative protein partners. These models and experiments also have not mapped out the specific amino acids responsible for these interactions or alveolin–alveolin compatibility.

Overall, this study provides a first interrogation into the molecular interactions and organizing principles that govern alveolin function and localization in the *Plasmodium falciparum* asexual blood stages. Together, our results are in line with previous evidence that the alveolin domain is a key driver of filament formation and recruitment. Specifically, our data provide evidence that filament formation and recruitment are linked. All of the recruitment we observed to be mediated by the alveolin domain was traced back to alveolin–alveolin interactions. This leaves the question of how the alveolins are recruited to their specific compartments when those are devoid of alveolins and whether the alveolin domain mediates that recruitment as well by interacting with other protein partners. While in silico modeling provides some clues as to the conformation these proteins might adopt, much remains to be learned about alveolin structure.

## 3. Materials and Methods

### 3.1. Accession Numbers.

The accession numbers for the genes analyzed in this study are as follows: *Pf*IMC1g (*PF*3D7_0525800), *Pf*IMC1c (*PF*3D7_1003600), *Pf*IMC1f (*PF*3D7_1351700), and *Pf*IMC1e (*PF*3D7_0304100).

### 3.2. Plasmid Construction [40].

All primer and gene block sequences are listed in Supporting Information 2: Table S1.

### 3.3. pAK60, 61, and 64 (Guide RNA Plasmids Targeting Pfbleb Locus).

The guides were annealed and ligated into BpiI-digested pRR216 (SpCas9 expression plasmid) to construct pAK60 (oJDD7004/7005), pAK61 (oJDD7006/7007), and pAK64 (oJDD5485/5486).

### 3.4. pAK86 (PfIMC1g Full Length in Trans Construct).

The *Pfbleb* 5′ homology region (HR) and 3′ HR region were amplified from *Pf*3D7 genomic DNA with oJDD6995/6996 and oJDD6997/6998, respectively. Codon-altered *Pf*IMC1g flanked by inverse *loxP511* and *loxN* sites and fused to a 1xV5 tag was amplified with oJDD7893/7895 and oJDD7893/7894. pGEM backbone was amplified from pCJM17 with oJDD6994/7003. Fragments were assembled with the Golden Gate BsaI-HF v2 Assembly Kit (NEB).

### 3.5. pAK90 (PfIMC1g Alveolin Domain Only in Trans Construct).

The *Pfbleb* 5′ and 3′ HR regions were amplified from pAK87 with oJDD7393/8008. Codon-altered *Pf*IMC1g alveolin domain, flanked by inverse *loxP511* and *loxN* sites and fused to an smV5 tag, was amplified with oJDD8006/8007. Fragments were assembled with the Golden Gate BsaI-HF v2 Assembly Kit (NEB).

### 3.6. pAK88 (PfIMC1g With PfIMC1e Alveolin Domain in Trans Construct).

The backbone containing *Pfbleb* 5′ and 3′ HR regions and the IMC1g N and C term were amplified from pAK86 with oJDD7924/7925. Codon-altered *Pf*IMC1e alveolin domain flanked by inverse *loxP511* and *loxN* sites and fused to a V5 tag was amplified with oJDD7926/7927 from pAK87. Fragments were assembled with the Golden Gate BsaI-HF v2 Assembly Kit (NEB).

### 3.7. pAK87 (PfIMC1e Full Length in Trans Construct).

The backbone containing *Pfbleb* 5′ and 3′ HR regions was amplified using oJDD7393/7433. Codon-altered *Pf*IMC1e flanked by inverse *loxP511* and *loxN* sites and fused to an smV5 tag was amplified with oJDD7891/7892 from pPB23. Fragments were assembled with the Golden Gate BsaI-HF v2 Assembly Kit (NEB).

### 3.8. pAK91 (PfIMC1e Alveolin Domain Only in Trans Construct).

The backbone containing *Pfbleb* 5′ and 3′ HR regions was amplified from pAK87 using oJDD7393/8008. Codon-altered *Pf*IMC1e alveolin domain flanked by inverse *loxP511* and *loxN* sites and fused to an smV5 tag was amplified with oJDD8009/8010 from pAK87. Fragments were assembled with the Golden Gate BsaI-HF v2 Assembly Kit (NEB).

### 3.9. pAK89 (PfIMC1e With PfIMC1g Alveolin Domain in Trans Construct).

The backbone containing *Pfbleb* 5′ and 3′ HR regions as well as the N and C termini of IMC1e was amplified from pAK87 using oJDD7928/7929. Codon-altered *Pf*IMC1g alveolin domain flanked by inverse *loxP511* and *loxN* sites and fused to an smV5 tag was amplified with oJDD7930/7931 from pAK86. Fragments were assembled with the Golden Gate BsaI-HF v2 Assembly Kit (NEB).

### 3.10. pAK102 (PfIMC1e NCR Domain Only in Trans Construct).

The backbone containing *Pfbleb* 5′ and 3′ HR regions was amplified from pAK91 using oJDD7393/8008. Codon-altered *Pf*IMC1e NCR domain flanked by inverse *loxP511* and *loxN* sites and fused to an smV5 tag was amplified with oJDD8065/8066 from pAK87. Fragments were assembled with the Golden Gate BsaI-HF v2 Assembly Kit (NEB).

### 3.11. pAK103 (PfIMC1e Alveolin + NCR Domain in Trans Construct).

The backbone containing *Pfbleb* 5′ and 3′ HR regions was amplified from pAK91 using oJDD8068/8008. Codon-altered *Pf*IMC1e sequencing flanked by inverse *loxP511* and *loxN* sites and fused to an smV5 tag was amplified with oJDD8065/8067 from pAK87. Fragments were assembled with the Golden Gate BsaI-HF v2 Assembly Kit (NEB).

### 3.12. pPB23 (PfIMC1e^iKO^ With smV5 Tag and hDHFR Selectable Marker).

The *PfIMC1e* 5′ and 3′ HR regions were amplified from *Pf*3D7 genomic DNA with oJDD7684/7685 and oJDD7690/7691, respectively. Codon-altered *Pf*IMC1e was amplified from synthetic gene block GB76 (IDT) with oJDD7686/7687. The pGEM backbone, smV5 tag, and hDHFR selectable markers were amplified from pAK79 with oJDD7682/7683 and 7688/7689, respectively. Fragments were assembled with the Golden Gate BsaI-HF v2 Assembly Kit (NEB).

### 3.13. pAK101 (PfIMC1e^iKO^ With smV5 Tag and BSD Selectable Marker).

The backbone containing *PfIMC1e* 5′ and 3′ HR regions and codon-altered IMC1e flanked by loxP sites and fused to an smV5 tag were amplified from pPB23 using oJDD7690/8047. BSD selectable marker was amplified with oJDD8051/8050. Fragments were assembled with the Golden Gate BsaI-HF v2 Assembly Kit (NEB).

### 3.14. pPB24–26 (Guide RNA Plasmids Targeting PfIMC1e).

The guides were annealed and ligated into BpiI-digested pFN41 (SpCas9 expression plasmid with no selectable markers) to construct pPB24 (oJDD7692/7693), pPB25 (oJDD7694/7695), and pPB26 (oJDD6336/6337).

### 3.15. pPB77 (PfIMC1f With smHA Tag).

*PfIMC1f* 5′ and 3′ HR regions were amplified from pAK57 using oJDD8204/8205 and oJDD8208/8209, respectively. pGEM backbone and smHA cassette were amplified from pPB45 with oJDD8202/8203 and oJDD8206/8207, respectively. Fragments were assembled with the Golden Gate BsaI-HF v2 Assembly Kit (NEB).

### 3.16. pRR255 (PfPIC5 With smV5 Tag).

*PfPIC5* 5′, 3′ HR, and codon-altered regions were amplified from 3D7 gDNA (or synthesized gene block, GB107) using oJDD5321/5322, oJDD5319/5320, and oJDD5323/5324, respectively. The three pieces were combined using overlapping PCR with oJDD5321/5324 and cloned into an smV5-containing plasmid (pRR190). The guide RNA was cloned into pRR216 with annealed oJDD5333/5334.

### 3.17. pAK104–106 (Guide RNA Plasmids Targeting the 3′ End of PfIMC1f).

The guides were annealed and ligated into BpiI-digested pRR216 (SpCas9 expression plasmid) to construct pAK104 (oJDD6464/6465), pAK105 (oJDD6466/6467), and pAK106 (oJDD6468/6469).

### 3.18. pAK35 (PfIMC1g^iKD^ With HA Tag).

pAK34 [8] was digested using PspOMI and NcoI and ligated to annealed oJDD6208/6209.

### 3.19. pAK73 (PfIMC1c With 2HA Tag).

pAK67 was digested using XmaI and NcoI and ligated to annealed oJDD7266/7267.

### 3.20. pAK109 (PfIMC1c^iKD^ With smV5 Tag).

The backbone containing *PfIMC1c* 5′ and 3′ HR regions was amplified from pAK67 using oJDD8309/8310. The smV5 tag and 10xTet-TetRDOZI machinery were amplified with oJDD8311/8312 from pPG03. Fragments were assembled with the Golden Gate BsaI-HF v2 Assembly Kit (NEB).

### 3.21. The Following Plasmids Were Previously Described [8].

pBAM377 was used to knock out *Pf*IMC1f, using guide RNA plasmids pBAM465 and pBAM553. pAK67 was used to tag *Pf*IMC1c 2HA and the iKD machinery, using guide RNA plasmids pAK37, 38, and 39. pAK17 was used to tag *Pf*IMC1g with 2HA and flank *loxPint* around its coding sequence, using guide RNA plasmids pRR189 and pAK30.

### 3.22. Protein Alignment and Analysis.

Protein sequences were obtained from PlasmoDB, and their domains were analyzed using InterPro [28]. Alignments were carried out using ClustalW (MegaX), and diagrams were generated using ESPript 3. Repeats were identified using HHrepID using maximal number of MSA steps (3) and repeat family *p* value threshold e−2 [29, 30]. Protein structures were generated using AlphaFold.

### 3.23. Plasmodium Falciparum Culture.

Parasite cultures were maintained as previously detailed [8]. Briefly, 3D7(pfs47) DiCre strains were cultured in RPMI 1640 supplemented with 25 mM HEPES, 0.21% sodium bicarbonate, 50 mg/L hypoxanthine, 0.5% AlbuMAX, and 2 mM choline chloride. Parasites were maintained in human O+ erythrocytes at 4% hematocrit under shaking conditions at 37°C.

#### 3.23.1. Parasite Synchronization.

Parasites were synchronized using Percoll density centrifugation and/or 5% (w/v) sorbitol selection as previously described [8].

#### 3.23.2. Parasite Transfection.

Homology-directed repair plasmid DNA (20 *μ*g) was linearized and mixed with Cas9-targeting plasmids (20 *μ*g each) and resuspended in 82 *μ*L buffer P3 (Lonza) with 18 *μ*L supplement solution (Lonza). Then, 10–30 *μ*L of packed schizonts were mixed and electroporated with the Lonza Nucleofector 4D. Electroporated schizonts were mixed with 200 *μ*L of packed RBCS in 1 mL of complete media and incubated at 37°C for 45 min before being transferred to a culture dish with complete media and 200 *μ*L of packed RBCs. Parasites were cultured under drug pressure starting at 24 h posttransfection. The integration of each targeting construct was confirmed by a PCR. Individual transgenic clones were obtained by a limiting dilution.

#### 3.23.3. Washout of ATc for Protein Knockdown.

The depletion was achieved by washing a tightly synchronized ring culture three times in ATc-free RPMI. The washed culture was then divided into two dishes with (or without) 500 nM ATc.

#### 3.23.4. Depletion by Inducible Knockout.

A tightly synchronized ring culture was divided into two dishes with 100 nM RAPA or equivalent volume of DMSO control.

### 3.24. Reagents and Antibodies

#### 3.24.1. Small Molecules.

Small molecules used in this study include WR99210 (Jacobus Pharmaceuticals; 2.5 nM working concentration), ATc (Cayman chemical, 0.5 *μ*M working concentration), and RAPA (Sigma, 100 nM working concentration).

#### 3.24.2. Primary Antibodies.

Commercially available antibodies include Sigma (rat anti-HA [clone 3F10]), Bio-Rad (mouse anti-V5), and Immunology Consultant Laboratories (rabbit anti-V5, RV5–45A-Z). Other antibodies were kindly provided by Julian Rayner at Cambridge Institute for Medical Research (rabbit anti-*Pf*GAP45) [41] and by Anthony Holder at MRC National Institute for Medical Research (mouse anti-*Pf*MSP1, clone 1E1) [42]. Rabbit anti-*Pf*IMC1g has been described previously [8].

#### 3.24.3. Secondary Antibodies.

All secondary antibodies for immunofluorescence and western blots were obtained from Thermo Fisher and LI-COR, respectively.

### 3.25. Genomic DNA Extraction.

Parasite genomic DNA was isolated from 5 to 10 mL of schizont-stage culture using the EZ-10 Spin Column Blood Genomic DNA Miniprep Kit (Bio Basic).

### 3.26. Growth Assays.

Flow cytometry–based growth assays were performed as previously described [8]. Briefly, each strain was diluted to 0.25% parasitemia at 1% hematocrit; 100 *μ*L of culture was collected 2 and 4 days after plating, stained with SYBR green, and measured by flow cytometry. One hundred thousand RBCs were counted for each condition, and the parasite multiplication rate was calculated as %RBCs infected at Day 4 divided by %RBCs infected at Day 2. Data represented as mean values of three independent biological replicates each with three technical replicates.

### 3.27. IFAs.

IFAs were performed as previously described [8]. Briefly, parasites were sedimented on poly-d-lysine-coated coverslips, fixed in prewarmed 4% PFA, and permeabilized with 0.1% Triton X-100. Coverslips were then blocked for 1 h at room temperature or overnight at 4°C, incubated in primary antibodies for 1 h at room temperature or overnight at 4°C, incubated in secondary antibodies for 45 min at room temperature, and mounted on a slide with VECTASHIELD Vibrance with DAPI.

Cells were visualized on a Zeiss LSM880 with Airyscan or Zeiss LSM900 with Airyscan 2 (Plan Apo 63×/1.4 Oil DIC III) for superresolution microscopy. Dilutions for primary antibodies were as follows: mouse anti-V5 between 1:200 and 1:1000, rabbit anti-*Pf*GAP45 1:5000, mouse anti-*Pf*MSP1 1:500, rat anti-HA 3F10 between 1:100 and 1:500, and rabbit anti-IMC1g 1:2500.

### 3.28. Western Blots.

Protocol was followed as previously described [43]. Parasites were isolated by lysing RBCs in 0.2% saponin in PBS with protease inhibitors (Pierce). Pelleted parasites were washed with PBS until the supernatant was clear; samples were then boiled in Laemmli buffer for 10 min. The equivalent of 1 mL of culture at 1% parasitemia was run on a 4%–20% mini-PROTEAN TGX gels (Bio-Rad) and transferred to nitrocellulose membranes. Membranes were blocked in Licor Odyssey blocking buffer, incubated with primary antibody, and then incubated in secondary antibodies diluted in blocking buffer. Membranes were scanned on a Licor Odyssey CLx imager system. The primary antibody dilutions are as follows: mouse anti-V5 (1:1000), rabbit anti-H3 (1:2500), and rat anti-HA (1:1000). The secondary antibody dilutions were all used at 1:10000.

## Supplementary Material

Supplemental Table 1 *Supporting Information 2*. Table S1: Oligonucleotides used in this study.

Supplemental Figures *Supporting Information 1*. Figure S1: Western blots of *in trans* constructs and *Pf*IMC1e/1f. All blots were probed with the indicated antibodies. Anti-H3 was used as the loading control with the expected size of 17 kDa. All molecular weights are indicated in kilodalton. (A) Diagram of the inducible *in trans* system. The construct is integrated into the *Pfbleb* locus in reverse orientation, and upon RAPA induction, the construct is irreversibly flipped in-frame. (B) Representative western blot of one of the *in trans* constructs in uninduced (DMSO) and induced (RAPA) conditions. Expected size of this particular construct is 57 kDa (arrow). (C) Negative control blot prepared with parental 3D7-DiCre parasites showing the absence of staining with both mouse anti-V5 and rat anti-HA. (D) The *Pf*IMC1g alveolin domain expressed in the *Pf*IMC1c^iKD^ parasites serves as a positive control because this *in trans* construct localizes properly to the IMC and does not depend on the presence of *Pf*IMC1c. The expected size is 73 kDa due to smV5-glmS-DD fusion. (E) The *Pf*IMC1e alveolin domain is expressed robustly despite being mislocalized. The expected size of the alveolin domain fused to smV5 tag is 61 kDa (arrow). (F) *Pf*IMC1g with its alveolin replaced with that of *Pf*IMC1e is expressed at diminished levels. The expected size with a single V5 tag is 36 kDa (arrow). (G) *Pf*IMC1e NCR domain is expressed robustly despite being mislocalized. The expected size with smV5 fusion is 57 kDa (arrow). (H) Endogenous *Pf*IMC1f is expressed at similar levels in the presence and absence of *Pf*IMC1e^iKO^. The expected size with smHA fusion is 190 kDa, though the protein is detected slightly higher. (I) In the absence of *Pf*IMC1f, endogenous *Pf*IMC1e^iKO^ is expressed robustly despite being mislocalized. Adding RAPA excises *Pf*IMC1e and results in no protein expression. The expected size with smV5 fusion is 106 kDa though the protein is detected slightly higher. Figure S2: Alignment of *Pf*IMC1g with 1e and 1c. (A) Alignment of the *Pf*IMC1g and *Pf*IMC1c amino acid sequence. (B) Alignment of the *Pf*IMC1g and *Pf*IMC1e amino acid sequence. Black segments indicate repeats identified within the alveolin domain. Gray segments indicate repeats identified outside the alveolin domain. Sequence alignment generated using ClustalW (MegaX), diagram generated using ESPript3. Repeats were identified using HHrepID. Figure S3: AlphaFold and Multimer models of alveolin structure. (A–C) AlphaFold and AlphaFold Multimer models of (A) *Pf*IMC1c alone, (B) *Pf*IMC1g alone, and (C) *Pf*IMC1g and *Pf*IMC1c together. (D–F) AlphaFold and AlphaFold Multimer models of (D) *Pf*IMC1e alveolin domain, (E) *Pf*IMC1e NCR region, and (F) full-length *Pf*IMC1e. (G–J) Predicted per-residue model confidence scores (pLDDT) of the top 5 ranked models and a cartoon representation of the top-ranked model. (G) Multimer models of *Pf*IMC1f and *Pf*IMC1e. (H) Multimer models of *Pf*IMC1g homodimer. (I) Multimer models of *Pf*IMC1g and *Pf*IMC1e heterodimer. (J) Multimer models of *Pf*IMC1e–1g hybrid protein where the alveolin domain of 1e has been replaced with that of 1g. Cyan = *α*-helix. Magenta = *β*-sheet. Figure S4. *Pf*IMC1g and *Pf*PIC5 colocalize throughout segmentation. IFAs are costained with *Pf*PIC5-smV5 (magenta), *Pf*IMC1g (green), and Hoechst for parasite DNA. The top row shows early segmentation, the middle row shows mid segmentation, and the bottom row shows late segmentation. Scale bars = 2 *μ*m.

Additional supporting information can be found online in the Supporting Information section.

## Data Availability

All data for this manuscript are presented in the article and the associated supporting files. Plasmids and parasite strains are available upon request.
