# Supplementary material for: Malaria Cytoskeletal Proteins Require Alveolin–Alveolin Interactions for Differential Localization: Recruitment and Organization of Alveolin Proteins
Source: Cell Microbiol. Author manuscript; Available in PMC 2025 Sep 30. (PMC12465100; doi:10.1155/cmi/4530231)
Supplement: Supplemental Figures — Supporting Information 1. Figure S1: Western blots of in trans constructs and PfIMC1e/1f. All blots were probed with the indicated antibodies. Anti-H3 was used as the loading control with the expected size of 17 kDa. All molecular weights are indicated in kilodalton. (A) Diagram of the inducible in trans system. The construct is integrated into the Pfbleb locus in reverse orientation, and upon RAPA induction, the construct is irreversibly flipped in-frame. (B) Representative western blot of one of the in trans constructs in uninduced (DMSO) and induced (RAPA) conditions. Expected size of this particular construct is 57 kDa (arrow). (C) Negative control blot prepared with parental 3D7-DiCre parasites showing the absence of staining with both mouse anti-V5 and rat anti-HA. (D) The PfIMC1g alveolin domain expressed in the PfIMC1ciKD parasites serves as a positive control because this in trans construct localizes properly to the IMC and does not depend on the presence of PfIMC1c. The expected size is 73 kDa due to smV5-glmS-DD fusion. (E) The PfIMC1e alveolin domain is expressed robustly despite being mislocalized. The expected size of the alveolin domain fused to smV5 tag is 61 kDa (arrow). (F) PfIMC1g with its alveolin replaced with that of PfIMC1e is expressed at diminished levels. The expected size with a single V5 tag is 36 kDa (arrow). (G) PfIMC1e NCR domain is expressed robustly despite being mislocalized. The expected size with smV5 fusion is 57 kDa (arrow). (H) Endogenous PfIMC1f is expressed at similar levels in the presence and absence of PfIMC1eiKO. The expected size with smHA fusion is 190 kDa, though the protein is detected slightly higher. (I) In the absence of PfIMC1f, endogenous PfIMC1eiKO is expressed robustly despite being mislocalized. Adding RAPA excises PfIMC1e and results in no protein expression. The expected size with smV5 fusion is 106 kDa though the protein is detected slightly higher. Figure S2: Alignment of PfIMC1g with 1e and 1c. (A) Alignm [file NIHMS2113050-supplement-Supplemental_Figures.pdf]

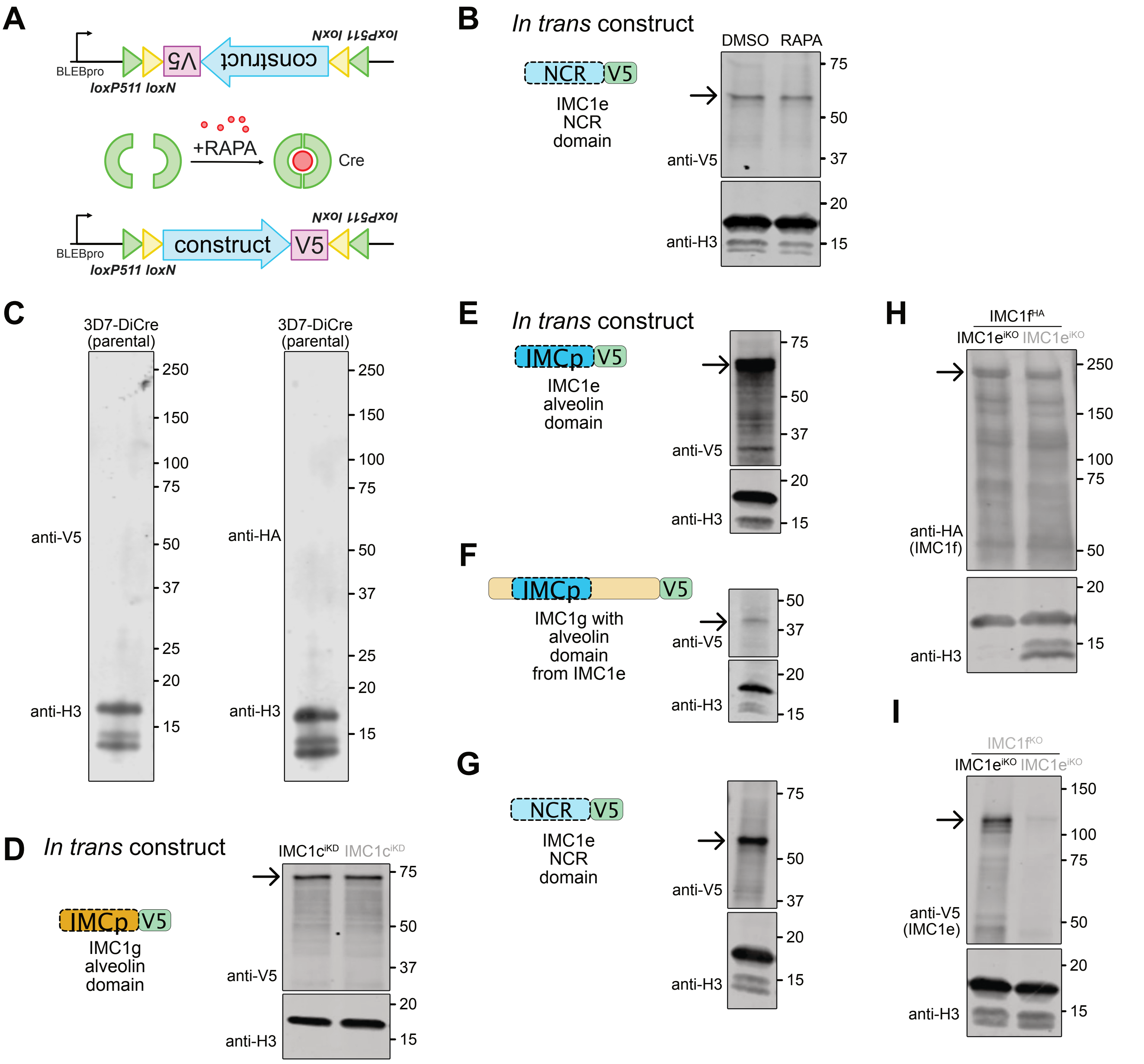

Supplemental Figure 1. Western blots of *in trans* constructs and *Pf*IMC1e/1f. All blots were probed with the indicated antibodies. Anti-H3 was used as the loading control with the expected size of 17 kDa. All molecular weights are indicated in kDa. **(A)** Diagram of the inducible *in trans* system. The construct is integrated into the *Pfbleb* locus in reverse orientation, and upon rapamycin induction, the construct is irreversibly flipped in-frame. **(B)** Representative western blot of one of the *in trans* constructs in uninduced (DMSO) and induced (RAPA) conditions. Expected size of this particular construct is 57 kDa (arrow). **(C)** Negative control blot prepared with parental 3D7-DiCre parasites showing absence of staining with both mouse anti-V5 and rat anti-HA. **(D)** *Pf*IMC1g alveolin domain expressed in the *Pf*IMC1c^{iKD} parasites serves as a positive control because this in trans construct localizes properly to the IMC and does not depend on the presence of *Pf*IMC1c. Expected size is 73 kDa due to smV5-glmS-DD fusion. **(E)** *Pf*IMC1e alveolin domain is expressed robustly despite being mislocalized. Expected size of the alveolin domain fused to smV5 tag is 61kDa (arrow). **(F)** ***Pf*IMC1**g with its alveolin replaced with that of *Pf*IMC1e is expressed at diminished levels. Expected size with a single V5 tag is 36kDa (arrow). **(G)** *Pf*IMC1e NCR domain is expressed robustly despite being mislocalized. Expected size with smV5 fusion is 57 kDa (arrow). **(H)** Endogenous *Pf*IMC1f is expressed at similar levels in the presence and absence of *Pf*IMC1e^{iKO}. Expected size with smHA fusion is 190 kDa, though the protein is detected slightly higher. **(I)** In the absence of *Pf*IMC1f, endogenous *Pf*IMC1e^{iKO} is expressed robustly despite being mislocalized. Adding rapamycin excises *Pf*IMC1e and results in no protein expression. Expected size with smV5 fusion is 106 kDa though the protein is detected slightly higher.

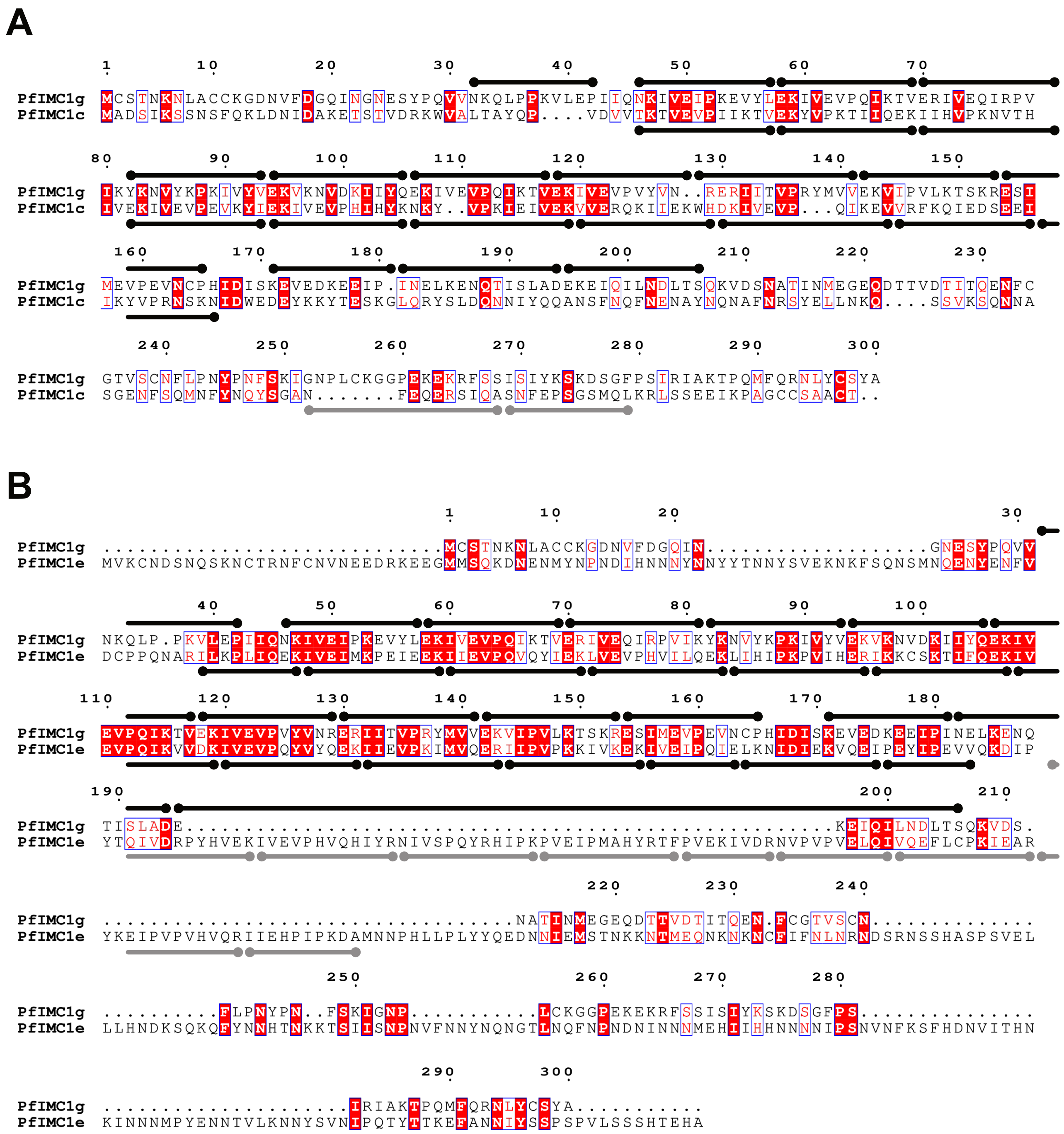

Supplemental Figure 2. Alignment of *Pf*IMC1g with 1e and 1c. (A) Alignment of the *Pf*IMC1g and *Pf*IMC1c amino acid sequence. **(B)** Alignment of the *Pf*IMC1g and *Pf*IMC1e amino acid sequence. Black segments indicate repeats identified within the alveolin domain. Grey segments indicate repeats identified outside the alveolin domain. Sequence alignment generated using ClustalW (MegaX), diagram generated using ESPript3. Repeats were identified using HHrepID.

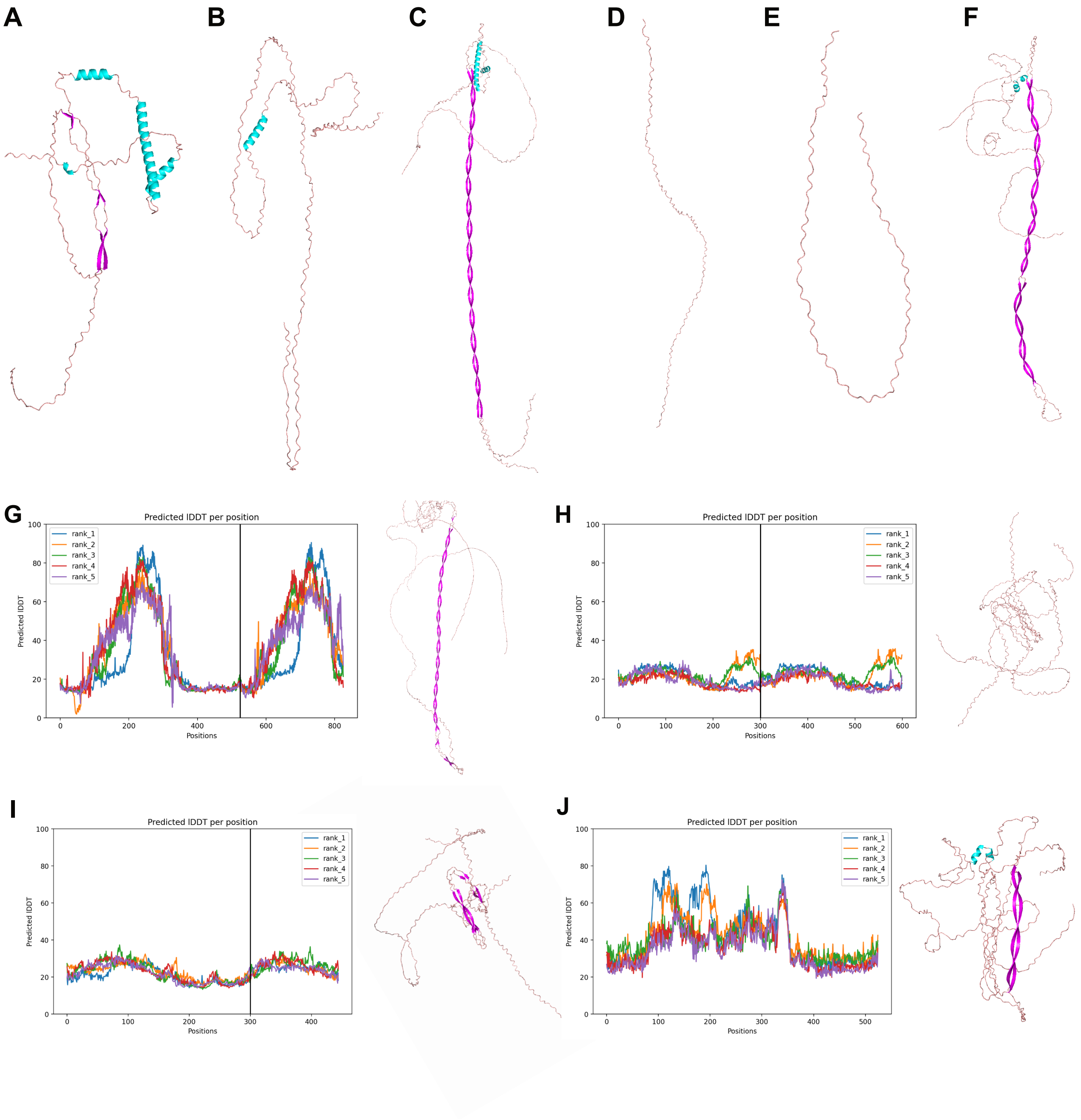

Supplemental Figure 3. AlphaFold and Multimer models of alveolin structure. (A-C) AlphaFold and Alphafold Multimer models of **(A)** *Pf*IMC1c alone, **(B)** *Pf*IMC1g alone, **(C)** *Pf*IMC1g and *Pf*IMC1c together. **(D-F)** AlphaFold and Alphafold Multimer models of **(D)** *Pf*IMC1e alveolin domain, **(E)** *Pf*IMC1e NCR region, (**F)** Full-length *Pf*IMC1e. **(G-J)** Predicted per-residue model confidence scores (pLDDT) of the top 5 ranked models and a cartoon representation of the top-ranked model. **(G)** Multimer models of *Pf*IMC1f and *Pf*IMC1e, **(H)** Multimer models of *Pf*IMC1g homodimer, **(I)** Multimer models of *Pf*IMC1g and *Pf*IMC1e heterodimer, **(J)** Multimer models of *Pf*IMC1e-1g hybrid protein where the alveolin domain of 1e has been replaced with that of 1g. Cyan = α-helix. Magenta = β-sheet.

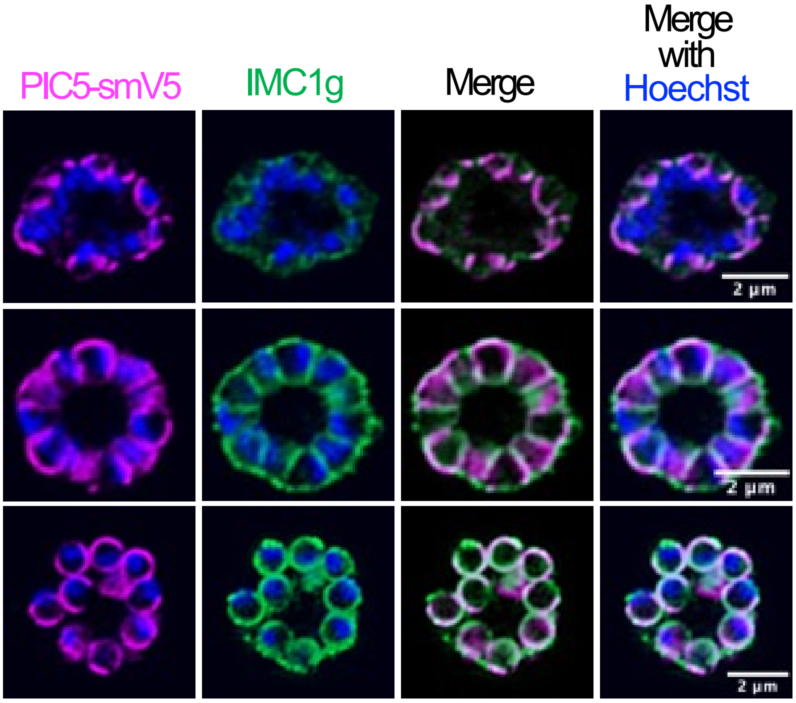

Supplemental Figure 4. *Pf*IMC1g and *Pf*PIC5 colocalize throughout segmentation. IFAs are co-stained with *Pf*PIC5-smV5 (magenta), *Pf*IMC1g (green), and Hoechst for parasite DNA. The top row shows early segmentation, the middle row shows mid segmentation, and the bottom row shows late segmentation. Scale bars = 2µm.
